# iOCT in der klinischen Anwendung: Korrelation von intraoperativer Morphologie und postoperativem Ergebnis bei Patienten mit durchgreifendem Makulaforamen

**DOI:** 10.1007/s00347-021-01527-w

**Published:** 2021-11-04

**Authors:** Julia Sabina Friedrich, Nathalie Bleidißel, Ali Nasseri, Nikolaus Feucht, Julian Klaas, Chris Patrick Lohmann, Mathias Maier

**Affiliations:** 1 Klinik und Poliklinik für Augenheilkunde, Klinikum rechts der Isar, Technische Universität München, Ismaningerstr. 22, 81675 München, Deutschland; 2 Smileeyes Augenklinik am Flughafen, München, Deutschland; 3 Augenklinik des Klinikums der Universität, Ludwig-Maximilians-Universität, München, Deutschland

**Keywords:** IOCT, Operative Bildgebung, Vitreoretinalchirurgie, Makulaforamen, SD-OCT, IOCT, Operative Imaging, Vitreoretinal Surgery, Macular Hole Surgery, SD-OCT

## Abstract

**Hintergrund:**

Die Beurteilung der intraoperativen Veränderung der Netzhautmorphologie, insbesondere des vitreoretinalen Überganges, ist mithilfe der intraoperativen optischen Kohärenztomografie (iOCT) möglich geworden.

**Ziel der Arbeit:**

Um die Bedeutung der intraoperativen Morphologie beim durchgreifenden Makulaforamen (MF) für das postoperative funktionelle Ergebnis zu evaluieren, wurde eine retrospektive, klinische Beobachtungsstudie durchgeführt.

**Material und Methoden:**

Die Netzhautmorphologie wurde in 32 Augen von 32 konsekutiven Patienten mit durchgreifendem Makulaforamen mittels iOCT zu verschiedenen Zeitpunkten während der Operation beobachtet. Die Veränderungen wurden anschließend mit dem postoperativen funktionellen Ergebnis korreliert.

**Ergebnisse:**

Nach Induktion der hinteren Glaskörperabhebung (HGA) reduzierte sich der Makulaforamen-Index (MHI) um −0,05 (*p* = 0,01), die basale Foramenbreite (FB) stieg um +99,4 μm (SD = 197,8 μm; *p* = 0,04). Die Verschlussrate betrug 100 % zum Zeitpunkt der ersten postoperativen Vorstellung nach im Mittel 73 Tagen, der postoperative Visus verbesserte sich signifikant (*p* < 0,05).

Es zeigte sich eine signifikant positive Korrelation von intraoperativer Morphologie und postoperativem Ergebnis zwischen einem niedrigen MHI und einem besseren postoperativen Visus (SKK = 0,50; *p* = 0,02), zwischen einer großen FB und einem besseren postoperativen Visus (SKK = 0,43; *p* = 0,05) sowie zwischen einer breiten Apertur nach HGA und einem größeren Visusanstieg postoperativ (SKK = 0,44; *p* = 0,03).

**Diskussion:**

Wir konnten eine Abflachung sowie eine Verbreiterung des MF durch Lösen der vitreoretinalen Zugkräfte beobachten. Aufgrund des Zusammenhangs zwischen einer großen intraoperativen FB mit einem besseren postoperativen Visus scheint die intraoperative Relaxierung der Netzhaut bedeutsam.

## Abstract (en)

**Background:**

Due to intraoperative optical coherence tomography (iOCT), observation of retinal morphological changes during surgery has become possible.

**Objective:**

To analyze the intraoperative morphology of full thickness macular holes (FTMH) and the correlation with the postoperative function, a retrospective, observational clinical study was performed analyzing 32 eyes of patients treated at the hospital of the technical university of Munich.

**Material and methods:**

Using iOCT in 32 eyes of 32 consecutive patients, the operative morphology was analyzed during surgery. These findings were then correlated with the postoperative visual outcome.

**Results:**

After posterior vitreous detachment (PVD) the macular hole index (MHI) decreased by −0.05 (*p* = 0.01) and the base diameter (BD) increased by +99.4 μm (SD = 197.8 μm; *p* = 0.04). Closure rate was 100% at the first visit after a mean time of 73 days and the postoperative best corrected visual acuity (BCVA) significantly improved (*p* < 0.05).

There were significant correlations between intraoperative morphology and postoperative results indicating a relation between low MHI and better postoperative BCVA (SCC = 0.50; *p* = 0.02), large BD and better postoperative BCVA (SCC = 0.43; *p* = 0.05) and large aperture after PVD and higher improvement of BCVA (SCC = 0.44; *p* = 0.03).

**Conclusion:**

Flattening and broadening of the FTMH occurred as a result of reduction of vitreoretinal traction. The significant correlation between a large operative BD and improved BCVA reveals the importance of intraoperative retinal relaxation.

## Hintergrund und Fragestellung

Durch die Integration des OCT-Gerätes in das Operationsmikroskop konnte die intraoperative Beobachtung mikrostruktureller Veränderungen der Netzhaut realisiert werden [3, 6, 9, 10, 12].

Neben Alter, Visus und MF-Größe als präoperative prognostische Faktoren [11, 19, 21] scheint der präoperative Makulaforamen-Index (MHI) relevant für das postoperative funktionelle Ergebnis zu sein [14].

Das Ziel dieser Studie war die Evaluation der Netzhautmorphologie während chirurgischer Manipulation bei Patienten mit MF mittels intraoperativer OCT (iOCT) und der Korrelation dieser Veränderungen mit dem postoperativen Visusergebnis.

## Studiendesign und Untersuchungsmethoden

Zur Evaluation des iOCT in der klinischen Routine wurde die intraoperative Netzhautmorphologie von 32 Augen von 32 konsekutiven Patienten analysiert. Aufgrund unvollständiger Daten waren 14 Patienten ausgeschlossen worden. Eingeschlossen wurden alle Patienten, die aufgrund eines idiopathischen, durchgreifenden Makulaforamens im Zeitraum von Mai 2015 bis Dezember 2016 iOCT-assistiert operiert wurden. Komorbiditäten wie Netzhautablösung, Glaskörperblutung, vaskuläre Erkrankungen, altersabhängige Makuladegeneration, Glaukom und jegliche zuvor durchgeführte Netzhautoperation inklusive der medikamenteninduzierten Vitreolyse mittels Ocriplasmin stellten Ausschlusskriterien dar.

Bestkorrigierter Visus, Linsenstatus und MF-Größe, gemessen an der Apertur, wurden präoperativ, im Zeitraum 3 Wochen bis 3 Monate postoperativ und nach mindestens 6 Monaten postoperativ evaluiert. Der Visus wurde standardmäßig in Dezimal erhoben, die Darstellung in dieser Arbeit erfolgt zur besseren Vergleichbarkeit umgerechnet in logMAR. Die MF-Apertur wurde präoperativ mithilfe des Heidelberg Eye Explorer (Heyex, Software Serial Number: H2E-18404-028-011, Heidelberg Engeneering, Heidelberg, Germany) vermessen.

Alle Patienten wurden mittels standardisierter transkonjunktivaler, nahtloser 23-Gauge-Pars-plana-Vitrektomie (ppV), iOCT- sowie färbungsgestütztem Membranpeeling der internen limitierenden Membran (ILM) (G-81005 Brilliant Peel ©; Fluoron, Geuder AG, Heidelberg, Germany) sowie Tamponade eines Luft-Gas-Gemisches (C3F8, 12 %) operiert. Die Analyse erfolgte anhand von intraoperativ gewonnenen iOCT-Volumenscans, aufgenommen mit dem OPMI Lumera 700 Mikroskop (Carl Zeiss Meditec AG, Jena, Germany) mit integriertem RESCAN 700 iOCT (SDOCT, 27000 A-Scans pro Sekunde, Wellenlänge 840 nm, A‑Scan Tiefe 2,0 mm, axiale Auflösung 5,5 µm [2]).

Die Operation wurde jeweils vom gleichen Ophthalmochirurgen (M. M.) durchgeführt, und die Auswertung der intraoperativen Morphologie bezog sich auf iOCT-Daten zu folgenden Zeitpunkten: zu Beginn der Operation, nach Induktion der hinteren Glaskörperabhebung (HGA) und nach ILM-Membranpeeling. Die Daten dieser iOCT-Scans bildeten die Grundlage für die retrospektive Auswertung.

Die retrospektive Vermessung der MF-Morphologie im intraoperativ gewonnenen iOCT-Volumenscan erfolgte mithilfe der Software ImageJ (freeware, National Institutes of Health, Bethesda, MD, USA).

**Figure 1:**
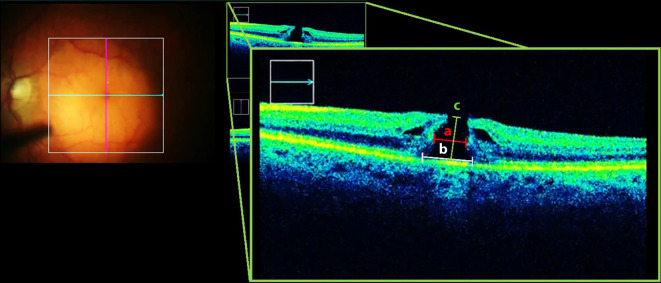

**Figure 2:**
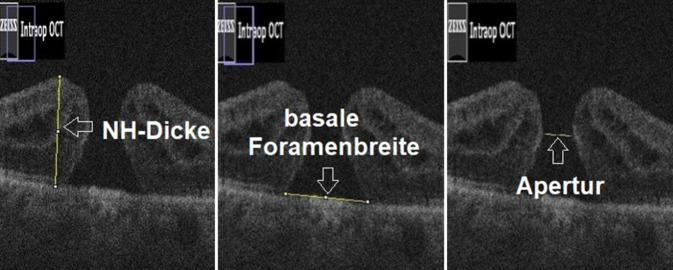

Die MF-Geometrie wurde dann anhand der Apertur, der basalen Foramenbreite (FB), der Netzhautdicke im Bereich der MF-Ränder sowie des MHI bestimmt. Die Netzhautmorphologie aus Sicht des Operateurs im iOCT ist in Abb. 1 dargestellt. Die retrospektive Vermessung der Parameter mithilfe der Software ImageJ ist in Abb. 2 veranschaulicht.

Die Apertur ist definiert als geringster Durchmesser des MF, gemessen auf Höhe der mittleren Netzhautschichten parallel zum retinalen Pigmentepithel (RPE) [4]. Die FB ist definiert als breitester Durchmesser des MF direkt über dem RPE gemessen, die Netzhautdicke im Bereich der MF-Ränder ist definiert als die größte Höhe des MF, gemessen von der ILM bis zum RPE, und der MHI errechnet sich aus dem Verhältnis der Netzhautdicke und der basalen Foramenbreite [14].

Die statistische Auswertung wurde mit der Software IBM SPSS Statistics Version 22 (IBM, New York, USA) auf einem Signifikanzniveau von *p* = 0,05 durchgeführt.

Es besteht ein Zulassungsvotum der Ethikkommission der TU München.

## Ergebnisse

Die Geschlechterverteilung innerhalb der 32 Patienten zeigte ein Verhältnis von annähernd 3,5:1 mit 25 (78 %) weiblichen und 7 (22 %) männlichen Patienten. Das durchschnittliche Alter betrug 69 Jahre (SD = 8,4 Jahre).

Der mittlere Visus betrug präoperativ 0,61 logMAR (SD = 0,25 logMAR) mit einer Pseudophakierate von 11 (34 %) Patienten. Die mittlere MF-Größe, gemessen an der Apertur, betrug präoperativ 332,6 µm (SD = 152,1 µm) mit einer Größenverteilung in kleines MF (< 250 µm – 34,5 %, *n* = 11), mittelgroßes MF (250–400 µm – 31 %, *n* = 10) und großes MF (> 400 µm – 34,5 %, *n* = 11) von jeweils annähernd einem Drittel [4].

Bei 8 (25 %) Patienten konnte im präoperativen SD-OCT eine vitreomakuläre Adhäsion, bei 12 (38 %) Patienten eine vitreopapilläre Adhäsion und bei 11 (34 %) Patienten eine komplette hintere Glaskörperabhebung nachgewiesen werden. Bei einem (3 %) Patienten konnte der vitreoretinale Übergang im präoperativen SD-OCT aufgrund mangelnder Bildqualität nicht beurteilt werden.

Nach HGA zeigten sowohl die Apertur (MW: −24,1 µm; SD = 97,8 µm; *p* = 0,27) als auch die Netzhautdicke im Bereich der MF-Ränder (MW: −19,6 µm; SD = 72,2 µm; *p* = 0,24) eine Abnahmetendenz. Die FB nahm nach HGA signifikant um +99,4 µm zu (SD = 197,8 µm; *p* = 0,04), und der MHI reduzierte sich signifikant um −0,45 (Minimum = −1,7; Maximum = +0,1; *p* = 0,01). Die Reduktion des MHI stellt eine Abnahme der Foramenhöhe in Relation zur basalen Foramenbreite dar.

Nach ILM-Membranpeeling zeigten sowohl die Apertur (MW: −40,5 µm; SD = 117,8; *p* = 0,14) als auch die FB (MW: −56,8 µm; SD = 369,6 µm; *p* = 0,52) eine Reduktionstendenz. Die Netzhautdicke im Bereich der MF-Ränder (MW: +24,7 µm; SD = 62,5 µm; *p* = 0,11) sowie der MHI (MW: +0,02; SD = 0,13; *p* = 0,53; t‑Test) zeigten eine Zunahmetendenz, was eine Zunahme der Foramenhöhe im Vergleich zur basalen Foramenbreite widerspiegelt.

**Figure 3:**
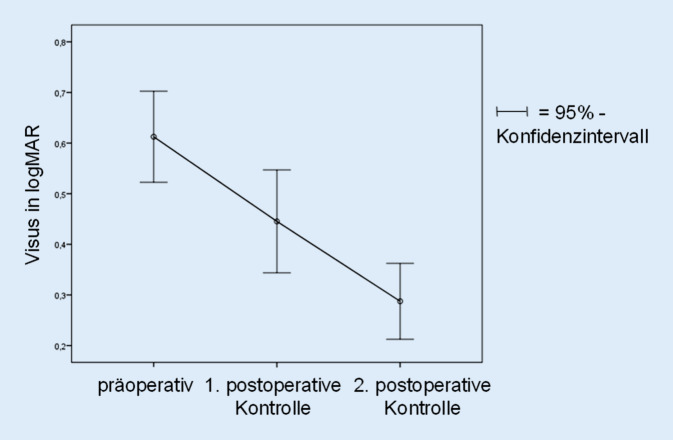

Die erste postoperative Datenerhebung erfolgte nach einer mittleren Zeit von 73 Tagen (SD = 63 Tage), und zu diesem Zeitpunkt zeigte sich eine MF-Verschlussrate von 100 %. Die Zeitspanne zwischen Operation und zweiter postoperativer Datenerhebung betrug im Mittel 297 Tage (SD = 118 Tage). Der Visus zum Zeitpunkt der ersten postoperativen Datenauswertung betrug 0,45 logMAR (SD = 0,28 logMAR) und zeigte sich damit im Vergleich zum präoperativen Visus (0,61 logMAR; SD = 0,25 logMAR) signifikant besser (−0,16 logMAR; SD = 0,28; *p* < 0,01). Der Visus zum Zeitpunkt der zweiten postoperativen Auswertung betrug 0,29 logMAR (SD = 0,21 logMAR) und war damit ebenfalls signifikant besser als der präoperative Visus (−0,32 logMAR; SD = 0,27; *p* < 0,01). Der Visusverlauf ist in Abb. 3 dargestellt.

Die Korrelation von intraoperativen Morphologieparametern (Apertur, Netzhautdicke im Bereich der MF-Ränder, FB, MHI) und Visus zum Zeitpunkt der ersten postoperativen Datenauswertung (73 Tage) ergab keine signifikanten Zusammenhänge.

Die Zusammenhänge von intraoperativer Morphologie und Visus zum Zeitpunkt der zweiten postoperativen Datenauswertung sind in Tab. 1 dargestellt.

**Table 1:** 

	Zu Beginn der Operation		Nach Induktion der HGA		Nach ILM-Membranpeeling	
	SKK	Signifikanz	SKK	Signifikanz	SKK	Signifikanz
Visus – Apertur	−0,34	*p* = 0,11	−0,15	*p* = 0,49	−0,25	*p* = 0,26
Visus – basale Foramenbreite	−0,11	*p* = 0,61	−0,16	*p* = 0,48	*−0,43*	*p* *=* *0,05*
Visus – Netzhautdicke	0,26	*p* = 0,24	0,28	*p* = 0,20	0,14	*p* = 0,53
Visus – MHI	0,20	*p* = 0,35	0,20	*p* = 0,35	*0,50*	*p* *=* *0,02*

*SKK* Spearman-Korrelationskoeffizient

Die Analyse in Bezug auf die relative postoperative Visusveränderung im Vergleich zum präoperativen Visus zeigte eine signifikante Korrelation zwischen einer breiten Apertur nach HGA und einer größeren Visusverbesserung postoperativ in unserer Kohorte von 32 Augen (SKK = 0,44; *p* = 0,03) (Tab. 2).

**Table 2:** 

	Nach Induktion der HGA	
	SKK	Signifikanz
Visusverbesserung – Apertur	*0,44*	*p* *=* *0,03*
Visusverbesserung – basale Foramenbreite	0,20	*p* = 0,37
Visusverbesserung – Netzhautdicke	0,23	*p* = 0,30
Visusverbesserung – MHI	−0,19	*p* = 0,38

*SKK* Spearman-Korrelationskoeffizient

**Figure 4:**
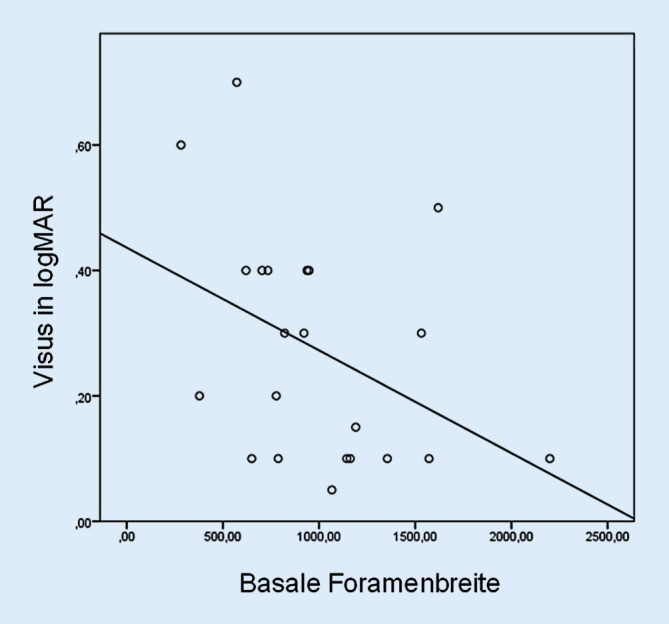

**Figure 5:**
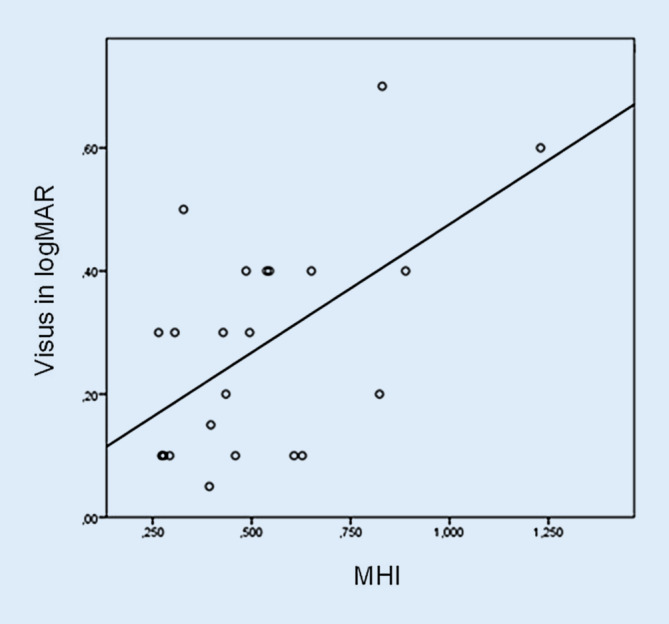

Des Weiteren zeigten sich eine signifikante Korrelation zwischen einer großen basalen Foramenbreite nach ILM-Membranpeeling und einem besseren postoperativen Visus (SKK = −0,43; *p* = 0,05) (Abb. 4) sowie eine signifikante Korrelation zwischen einem niedrigen MHI nach ILM-Membranpeeling und einem besseren postoperativen Visus (SKK = 0,50; *p* = 0,02) (Abb. 5). Dies weist darauf hin, dass ein niedriger intraoperativer MHI, welcher eine große FB im Verhältnis zur Netzhautdicke im Bereich der MF-Ränder widerspiegelt, ein positiver prognostischer Marker für den postoperativen Visus ist (Tab. 3).

**Table 3:** 

	Signifikanz	MHI < 0,5	MHI ≥ 0,5
MHI zu Beginn der Operation	*p* = 0,90	*n* = 12	*n* = 11
MHI nach Induktion der HGA	*p* = 0,93	*n* = 16	*n* =8
MHI nach ILM-Membranpeeling	*p* = 0,06	*n* = 14	*n* =9

## Diskussion

Unsere Beobachtungen zeigen eine signifikante Zunahme der FB und eine signifikante Abnahme des MHI nach HGA. Die Breitenzunahme und Abflachung des MF interpretieren wir als Ausdruck einer Entspannung der Netzhaut aufgrund der Reduktion vitreoretinaler Zugkräfte. Bezogen auf die Apertur und die FB verkleinert sich das MF nach ILM-Membranpeeling am ehesten durch das Nachlassen tangentialer Zugkräfte der ILM auf die Netzhaut. In unserer Kohorte von 32 Augen zeigte sich ein signifikanter Zusammenhang zwischen einer intraoperativ großen FB und einem besseren Visusergebnis sowie zwischen einem intraoperativ niedrigen MHI und einem besseren Visusergebnis. Wir interpretieren ein intraoperativ verbreitetes und abgeflachtes MF als einen guten prognostischen Faktor.

Sowohl der Zusammenhang einer großen FB nach ILM-Membranpeeling als auch einer breiteren Apertur nach HGA mit einem besseren postoperativen Visus zeigt, dass die intraoperative Reduktion von zuvor bestehenden Zugkräften auf den vitreoretinalen Übergang und die damit verbundene Entspannung der Netzhaut sehr bedeutsam für das postoperative funktionale Ergebnis ist. Bereits 2014 beobachteten Ehlers et al. einen signifikanten Zusammenhang zwischen der intraoperativen Veränderung der basalen Foramenbreite nach ILM-Membranpeeling und der MF-Verschlussrate (*p* = 0,01) [8]. Meyer et al. beschrieben 2017 die Lösung der Verzahnung von Photorezeptoren und RPE durch subretinale Applikation von Flüssigkeit. Die so induzierte Mobilität der Netzhaut fördert den MF-Verschluss [18]. Ehlers et al. beschrieben eine intraoperative Expansion zwischen EZ und RPE als zunehmende Netzhautmobilität [7]. In Analogie hierzu könnten eine intraoperative Zunahme der Apertur und der FB ebenfalls Ausdruck der Netzhautmobilität sein. Durch reduzierte retinale Adhärenz werden die Readaptation der Foramenränder und ein zügiger Heilungsverlauf gefördert [16].

Kusuhara et al. wiesen 2004 bei Patienten mit einem präoperativen MHI ≥ 0,5 einen besseren postoperativen Visus nach als bei Patienten mit einem präoperativen MHI < 0,5 (*p* = 0,03) [14]. Zu einem ähnlichen Ergebnis kamen wir in einer Langzeitnachverfolgung von Patienten mit MF, welche mithilfe der Inverted-Flap-Membranpeeling-Technik operiert wurden [1]. Beide Arbeiten verwendeten zur statistischen Auswertung die präoperativen MHI-Daten. Im Gegensatz dazu zeigten die Ergebnisse der vorliegenden Arbeit eine signifikante Korrelation zwischen einem niedrigen intraoperativen MHI und einem besseren postoperativen Visus (*p* = 0,02). Der präoperative MHI scheint eine andere Aussagekraft zu haben als der intraoperativ erhobene MHI. Die intraoperative Veränderung des MHI könnte ein weiteres Maß für die durch Meyer et al. beschriebene Mobilität der Netzhaut sein [18].

Eine Limitierung der vorliegenden Arbeit ist das retrospektive Studiendesign. Des Weiteren könnte das postoperative Visusergebnis von der Dauer der Erkrankung beeinflusst sein [13]. Da die Zeitspanne zwischen Erstauftreten von Symptomen und dem Operationszeitpunkt nicht erfasst wurde, war eine Analyse diesbezüglich retrospektiv nicht möglich.

Bei 6 (19 %) Patienten erfolgte intraoperativ ein ILM-Membranpeeling mit sog. Inverted-Flap-Technik [15], da es sich um Patienten mit sehr großem MF handelte. Das Ziel unserer Arbeit bestand in der Auswertung der klinischen Anwendung des iOCT anhand der Beobachtung der intraoperativen Morphologie. Unserer Ansicht nach wird die Analyse des Stellenwertes des iOCT nicht maßgeblich durch diese Operationstechnik beeinflusst, da der ILM-Flap bei der sog. Cover-Technik [19] auf der Netzhaut positioniert, jedoch nicht in das MF hineingedrückt wird.

Präoperativ waren 11 (34 %) Patienten pseudophak. Dieser Anteil stieg zum Zeitpunkt der ersten postoperativen Analyse auf 17 (53 %) Patienten und zum Zeitpunkt der zweiten postoperativen Analyse auf 24 (75 %) Patienten an. Eine negative Beeinflussung des postoperativen Visus durch die mögliche Kataraktentwicklung bei den 8 (25 %) phaken Patienten mit einem mittleren Alter von 69 Jahren kann nicht ausgeschlossen werden. Die retrospektive Auswertung der Daten ließ keine Schweregradeinstufung der Katarakt zu.

Unsere Kohorte zeigte eine gleichmäßige Größenverteilung des MF (klein, mittel, groß). Dennoch muss in Betracht gezogen werden, dass Patienten mit einem großen MF präoperativ einen geringeren Visus aufweisen als Patienten mit einem kleinen Makulaforamen. Das Visusverbesserungspotenzial ist bei Patienten mit großem MF demnach, statistisch gesehen, größer als bei Patienten mit kleinem Makulaforamen, wobei ein präoperativ großes Makulaforamen als negativer prognostischer Marker gilt.

Ein iOCT-integriertes Messinstrument zur Analyse der MF-Morphologie in Echtzeit stand dem Operateur nicht zur Verfügung.

Unsere Arbeit zeigt die Möglichkeit der Visualisierung von intraoperativen Veränderungen der Netzhautmorphologie mittels iOCT in der klinischen Anwendung. Eine intraoperative Anpassung der therapeutischen Strategie ist dadurch möglich geworden [5, 15, 17, 20].

## Fazit für die Praxis

- Dank der iOCT wird die intraoperative Beobachtung der Netzhautmorphologie und deren Veränderung bei Patienten mit durchgreifendem Makulaforamen möglich.
- Während der Operation zeigt sich eine Entspannung der Netzhaut, welche sich in einer Breitenzunahme und einer Höhenabnahme des Makulaforamens darstellt.
- Eine mobile Netzhaut am Ende der Operation steht in Zusammenhang mit einem besseren postoperativen Visus.
